# The Effect of a Mutation in the Thyroid Stimulating Hormone Receptor (TSHR) on Development, Behaviour and TH Levels in Domesticated Chickens

**DOI:** 10.1371/journal.pone.0129040

**Published:** 2015-06-08

**Authors:** Anna-Carin Karlsson, Frida Svemer, Jonas Eriksson, Veerle M. Darras, Leif Andersson, Per Jensen

**Affiliations:** 1 IFM Biology, Division of Zoology, Linköping University, SE-581 83 Linköping Sweden; 2 Department of Medical Biochemistry and Microbiology, Uppsala University, Box 582, SE-751 23 Uppsala, Sweden; 3 Laboratory of Comparative Endocrinology, Department of Biology, Division of Animal Physiology and Neurobiology, KU Leuven, B-3000 Leuven, Belgium; CSIRO, AUSTRALIA

## Abstract

The thyroid stimulating hormone receptor (*TSHR*) has been suggested to be a “domestication locus” in the chicken, due to a strong selective sweep over the gene found in domesticated chickens, differentiating them from their wild ancestor the Red Junglefowl (RJF). We investigated the effect of the mutation on development (incubation time), behaviour and thyroid hormone levels in intercross chickens homozygous for the mutation (*d/d*), wild type homozygotes (*w/w*) or heterozygotes (*d/w*). This allowed an assessment of the effect of genotype at this locus against a random mix of RJF and WL genotypes throughout the rest of the genome, controlling for family effects. The *d/d* genotype showed a longer incubation time, less fearful behaviours, lower number of aggressive behaviours and decreased levels of the thyroid hormone T4, in comparison to the *w/w* genotype. The difference between *TSHR* genotypes (*d/d* vs. *w/*w) in these respects mirrors the differences in development and behaviour between pure domesticated White Leghorns and pure RJF chickens. Higher individual T3 and T4 levels were associated with more aggression. Our study indicates that the *TSHR* mutation affects typical domestication traits, possibly through modifying plasma levels of thyroid hormones, and may therefore have been important during the evolution of the domestic chicken.

## Introduction

Domestication is the process where animals adapt to a life with humans and it includes genetic as well as developmental changes [1]. The adaptation to a captive environment tends to modify a whole array of traits towards what has been termed “the domesticated phenotype”, where the domesticated animal differs from its wild ancestor in morphology, physiology, development and behaviour [2,3]. In chickens, the White Leghorn (WL, *Gallus gallus domesticus*), a domesticated chicken line selected for high egg production, differs from its wild ancestor, the Red Junglefowl (RJF, *Gallus gallus*) in a number of different traits such as size, colour and reproduction [2,4]. WL chickens also show some striking differences in behaviour in comparison to the RJF. As a response to potentially fearful stimuli WL expresses a lower level of fearful behaviours than RJF [5]. Furthermore, studies have shown that WL in general are less active, show a lower frequency of social interactions and use a more energy-conserving foraging strategy than RJF [2,6,7].

The human driven selection towards a “domesticated phenotype” has caused changes in allele frequencies at many loci [8]. In a study by [9], eight populations of domestic chickens as well as populations of RJF were resequenced to identify selective sweeps of favourable alleles, which may have had a prominent role during chicken domestication. The result showed that all sequenced domestic chickens shared a selective sweep at the locus for the thyroid stimulation hormone receptor (*TSHR*) and a missense mutation causing a non-conservative glycine to argenine substitution was suggested as candidate mutation for the *TSHR* sweep. Glycine at this position is conserved among all known vertebrate TSHR sequences and a bioinformatics analysis predicted the substitution to push the residue outwards from the membrane and hence influence ligand interaction [9]. The chicken was domesticated already 6000 B.C. [10] and because most domesticated chicken worldwide carry the mutant *TSHR* allele, it indicates that the mutation is old. In fact, it has recently been reported that the mutation was present at a high frequency already in chickens that were used in the Roman empire [11]. Thus, *TSHR* may be a domestication locus in chicken [9], although, so far, the phenotype caused by the mutation remains unknown.

The TSHR is located mainly at the surface of the thyroid follicle cells in the thyroid gland and serves as a binding site for thyroid stimulating hormone (TSH). TSH is released from the pituitary gland upon stimulation from hypothalamus via thyrotropin-releasing hormone and corticotropin-releasing hormone [12], and the binding of the TSH ligand to its receptor stimulates the synthesis and release of thyroid hormones (THs) from the thyroid gland into the blood stream. Thyroid hormones, triiodothyronine (T3) and thyroxin (T4), are known to have either direct or indirect effects on metabolism, gene regulation, growth, reproduction and pigmentation, which are all traits affected throughout the domestication process [13]. The effect of TH begins already during embryonic development. In precocial birds, as the chicken, the thyroid gland develops early in embryonic life and TH secretion starts at the end of the first week of incubation [14]. During the latter half of incubation, plasma TH levels increase rapidly with a clear peak, mainly in T4, during the perihatch period [15, 16]. THs are important in the process of hatching and antithyroid drugs injected into the egg have shown to delay hatching [17], while chickens treated with TH hatch earlier [18].

It is well established from studies on different animals that THs have an effect on behaviour as well. Treatment with methimazole, which inhibits TH biosynthesis, during incubation decreases T3 plasma levels in chickens, and causes slower aggregation behaviour, reduces social attachment and decreases vocalization activity in chickens. THs have also been shown to affect behaviour in dogs, where behavioural problems have been associated with both higher and lower levels of total T4 than normal [19,20]. Furthermore, male Sprague-Dawley rats have been shown to decrease their social dominance behaviour after ligation of the sciatic nerve causing a decrease in mean T3, T4 and free T4 plasma levels [21], and hypothyroidism in male mice produces a mild anxiogenic effect [22].

Mutations in *TSHR* are known to affect TH levels but there are few studies exploring a direct effect on behaviour. One example though, is the *TSHR* ^hyt^ mouse where a point mutation causes a defective *TSHR* and hypothyroidism. The mutation has been reported to have a direct effect on behaviour, where about 25% of the homozygous animals show a spontaneous, asymmetrical circling behaviour [23]. However, it is still an open question if the widespread mutation in the *TSHR* gene of domesticated chickens reported by [9] has clear phenotypic effects relating to domestication traits and behaviour. The strong selective sweep at *TSHR* strongly suggests that one or more mutations at this locus may have contributed significantly to the evolution of the domestic chicken and may affect domestication-related traits seen in todays modern breeds. At present it is unknown how the mutation in the *TSHR* gene affects the receptor affinity and signal transduction, and therefore it is difficult to predict the biological outcome of the mutation. Therefore, the aim of this study was to explore if the missense mutation in the *TSHR* gene affects development (incubation time), behaviour and TH levels in chicken.

The animals used in this study were generated from a Locus Controlled Advanced Intercross Line (LAIL), in which the genotype of a locus under interest is kept constant and controlled, while the genotypes in the rest of the genome varies randomly due to random recombinations between the original parental chromosomes [24]. The chickens were bred from an F8-generation of an advanced intercross line between RJF and WL and were either homozygous for the wild type *TSHR* allele *w/w*, heterozygous *w/d* or homozygous for the mutant allele *d/d*. With this approach, we could study the effect of the genotype at this locus against a random background present in the RJF x WL LAIL achieved by accumulating recombinations in the previous eight generations, while controlling for family effects since the parents were all heterozygous for the targeted mutation. We recorded the behaviour in a number of different test situations and collected blood for thyroid hormone analysis in order to find consistent differences between *TSHR* genotypes. Groups of pure-bred RJF and WL were bred and used as control groups in some of the behavioural tests. We hypothesized that animals with different *TSHR* genotypes would differ in behaviour and hormone levels, and that the differences between *TSHR w/w* and *TSHR d/d* would resemble the differences between pure RJF and WL.

## Materials and Methods

### Animals

The study was approved by Linköping local Ethical committee of The Swedish National Board for Laboratory Animals (approval no. Lkp 85–07).

The chickens used were offspring from the F8 generation of an advanced intercross between White Leghorn and Red Junglefowl. The White Leghorn line used for this cross (SLU 13) has a long history of selection for egg production traits and originated from the Scandinavian selection and crossbreeding experiment [25]. The Red Junglefowl originated from a Swedish zoo population; more details about the original animals used for the intercross can be found in [7].

The chickens were pedigree hatched and bred from 7 families, where all parental birds were heterozygous *w/d* at the *TSHR* locus. Two batches with a total of 151 chickens were hatched. Batch 1 consisted of 49 females (*w/w*: 16, *w/d*: 26, *d/d*: 7) and 20 males (*w/w*: 4, *w/d*: 9, *d/d*: 7) and batch 2 of 36 females (*w/w*: 9, *w/d*: 18, *d/d*: 9) and 46 males (*w/w*: 14, *w/d*: 19, *d/d*: 13). In the following, these birds are referred to as the “*TSHR* chickens”. Both batches were treated identically throughout the experiment. Because of varying constraints of time and space, different sample sizes were used in each of the different tests, as specified below.

The chickens were hatched in automated incubators (Masalles 25 DIGIT) at the”Kruijt” hatchery at Linköping University, Sweden. At hatching, the chickens were weighed, marked with wing-tags and vaccinated against Marek’s disease. From day 1, the chickens were kept in pens measuring 1.4x0.7x1.7 m (length×width×height) with *ad libitum* access to food (commercial chicken food) and fresh water. Room temperature was maintained at 25°C and the light (5 lux) was kept on a 12:12 h light:dark cycle. At 5 days of age, blood samples were collected from all individuals for genotyping (see below).

At an age of 6 weeks, all chickens were moved to the “Wood-Gush” research chicken house, 10 km outside of Linköping. The chickens were divided by sex and housed in two identical pens measuring 3.0×2.5×3.0 m (l×w×h) with full visual and auditory contact between the pens. The pens contained food and water *ad libitum*, perches, nest boxes and wood shavings on the floor. The chickens were kept on a 12:12 h light:dark cycle and the room temperatute was 20°C.

Pure-bred RJF (26 birds) and WL (69 birds) chickens from the parental lines (described above) were also used in some of the behavioural tests in this study. These birds were tested at a young age and hence no sex determination was possible. The purebred chickens received the same treatment from hatching onwards as the *TSHR* chickens.

### Genotyping

Blood samples were diluted 100 times prior to PCR amplification. The *TSHR* genotypes were determined using the Phusion Blood Direct PCR Kit (Thermo Fisher Scientific) and the PCR reactions were set up according to the manufacturer’s instructions. The primers used for amplification and sequencing were forward primer 5´- TGGTTATCATGCTGGGAGGT-3´ and reverse primer 5´- GAGCCATGCACAGAAAGTCA-3´. The sequences were analyzed and edited with Codon Code Aligner (CodonCode, Dedham, MA)

### Behavioural tests

#### Incubation time

Time of incubation for each individual egg was measured for both *TSHR* and RJF/WL chickens (in total 235 birds). Before incubation started, two IR-cameras (KGuard Security OT401-4FW426A) were mounted inside each totally darkened hatcher. Each egg was placed in an individual transparent container covered with a piece of mesh, at day 19 of incubation, to avoid the chickens to mix after hatch. Recordings were made with 20 min time lapses, starting at day 20 of incubation, and lasting until all eggs had hatched. The time of hatching, defined as the whole chicken being visible to the camera, was analyzed from the videos. Incubation time was measured in minutes following the first egg that hatched. Chicks which had hatched were removed from the incubator every six hour during the incubation period. They were placed in pens according to the description above.

#### Open field

Both *TSHR* (N = 146) and RJF/WL (N = 95) chickens were exposed to an open field-test starting at the same age, 21 days, in order to measure general activity in a novel environment (see [26]for details regarding the test). The arena was made of plywood and measured 1.0x0.8 m with one start zone in the corner measuring 0.2x0.2 m. The bird was placed, in darkness, in the start zone and the test started when the light was turned on. The time spent in the start zone and the total distance moved was measured using the software Ethovision XP from Noldus.

#### Social reinstatement

The tendency of the chickens to reinstate social contact was measured at the same age for both *TSHR* (N = 146) and RJF/WL (N = 93) chickens starting at 28 days. (see [24] for details regarding the test). The arena was a straight corridor measuring 1.0x0.3 m divided into equally sized start zone, middle zone and social zone. The bird was placed, in darkness, in the start zone and the test started when the light was turned on. The time the chickens spent in the start zone and the total distance moved were measured using the software Ethovision XP from Noldus.

#### Fear of human

Starting at the age of 175 days the *TSHR* chickens (N = 90) were tested for fear responses to a human (see [26] for details regarding the test). The fear of human test has been performed in RJF and WL chickens in a previous study (see [5]), therefore no RJF/WL birds were included in this test. Each TSHR chicken was tested individually in a test arena measuring 1.5×0.5×0.5 m (l×w×h) divided into three equally sized zones: start zone, middle zone and human zone. Prior to test start, six chickens at a time were food and water deprived for 30–60 min while kept in a plastic box. Before the test started, one chicken was placed in the start zone for two min of habituation. A solid sliding door prevented the bird to access the arena before start and the test started when the door opened. The observer was sitting quietly outside the human zone facing the test arena with a hand containing standard chicken food placed in the center of the human zone. Five minutes of behavioural recording was conducted using 1/0 sampling with 10 s intervals. The behaviours freeze, lie and stand were recorded (see Table 1 for a full description of the behaviours) and pooled into one variable named “fearful behaviours”.

**Table 1:** Behaviours recorded in the fear of human and social dominance test.

Behavioural test/Categories	Behaviour	Description
Fear of human/Fearful behaviours	Freeze	Standing or lying immobile
	Lie	Lying on ground with eyes open
	Stand	Standing with eyes open
Social dominance/Aggressive behaviours	Aggressive peck	Gives a fast peck towards the other bird
	Attack	Runs towards the other peck and gives a pec
	Chase	Follows the other bird in an aggressive context
	Fight	Birds aggressively attack each other with claws and beaks
	Threat	Stiff body posture towards the other bird standing within 25 cm
	Raised hackle threat	Threat with hackles raised towards the other bird
	Threat with wing flap	Threats while flapping wings
	Walzing agonistic	Wals around the other bird with one wing lowered

#### Social dominance

The social dominance-test was conducted on the *TSHR* chickens (N = 42) starting at 189 days of age in order to measure dominance over a limited resource. The test was modified from previous studies [27–29] to suit our experimental design. No WL/RJF chickens were used in this test because of the big difference in size between the breeds. Body size affects social hierarchy where heavier animals are more dominant [30], which would predispose the heavier WL to be dominant over RJF. Moreover, WL are more aggressive directly after regrouping in comparison to RJF, but not over time [31]. One week prior to testing, the TSHR birds were habituated to food containers with mealworms in their home boxes. The chickens were tested in a test box measuring 1.0×1.0×1.7 m (l×w×h) made of three solid walls and a front of mesh to allow the observer full visual access. Two chickens of the same sex but different *TSHR* genotypes were tested simultaneously. Before starting, the test box was divided into two equal sized compartments with a solid wall to avoid physical and visual contact, and one animal in each compartment was habituated to the arena for 15 min. At the start of the test, the solid wall was removed and the chickens were allowed to interact freely, with one food container with meal worms in the arena. Only one chicken at a time could access the meal worms through a hole in a transparent plexiglass cover on top of the food container. The test lasted until one chicken had started to eat from the food container and the frequency of the behaviours aggressive peck, attack, chase, fight, raised hackle threat, threat, threat with wing flap and walzing agonistic (see Table 1 for a full description of the behaviours) were measured continuously and pooled together into one variable named “aggressive behaviours”. All chicken pairs were exposed to two consecutive tests with 30 min of separation with a solid wall and no access to meal worms in between, and all genotypes were tested against each other. Total number of interactions was limited by the genotype group with smallest number of individuals for each sex. From the two genotype groups with more individuals than the limiting group, chickens were randomly selected.

In order to minimize risks of injury and harm to the animals, a test termination criterion was set up to stop any test in which any of the animals were obviously severely stressed (crouching or hiding in a corner, consistently trying to escape) or if there was any blood shed from any of the involved birds. In this case, veterinary care was to be undertaken immediately. However, in no case did this happen, so no tests were interrupted.

### TH analyses

Total T4 and T3 levels in plasma were measured for the *TSHR* chickens (N = 114) at 70 days of age by an in-house RIA system [32] using a standard in hormone-free serum and results were expressed as picomole per ml plasma. The T3 RIA had a detection limit of 2 fmol and an intra-assay variability of 2.2%. The T4 RIA had a detection limit of 5 fmol and an intra-assay variability of 2.8%. For the T3 RIA cross-reactivity with T4 was 0.1–0.5%, whereas for the T4 RIA cross-reactivity with T3 was 3.5%. Unfortunately, thyroid hormone measurements in RJF and WL chickens were not possible due to practical issues.

### Statistics

For analysing genotype effects in the purebred and intercross chickens, we used Generalized Linear Models (SPSS v. 22.0). For the purebred, the model included only genotype as predictor. For TSHR-birds, genotype nested within family was used as predictor, together with sex, batch and the interaction of genotype x sex. The probability distribution used was “Normal” and the link function “Identity”, except for frequency of aggressive behaviors (a count variable), where we used Poisson distribution and Log link. For effects of genotype on aggressive behaviours, we could also not fit the family variable, since birds were tested in pairs from different families. We analysed whether there was any individual effect of either T3 or T4 levels on one hand, and frequency of aggression or fear of humans on the other, using a Generalized Linear Model with hormone levels nested within families, and including sex in the model. Significance levels were determined with the Wald Chi-squared test with adequate degrees of freedom. The Omnibus (Likelihood Ratio Chi-Square) test was used for determining the performance of the model versus the intercept, and this was deemed acceptable when the significance level was below 0.05. This was the case for all results reported here. Results are reported as estimated marginal means with their standard errors. Tukey’s post hoc test was used for pair-wise comparisons of significant effects. Effects of batch were mostly not significant, but were still kept in all models.

## Results

**Fig 1:**
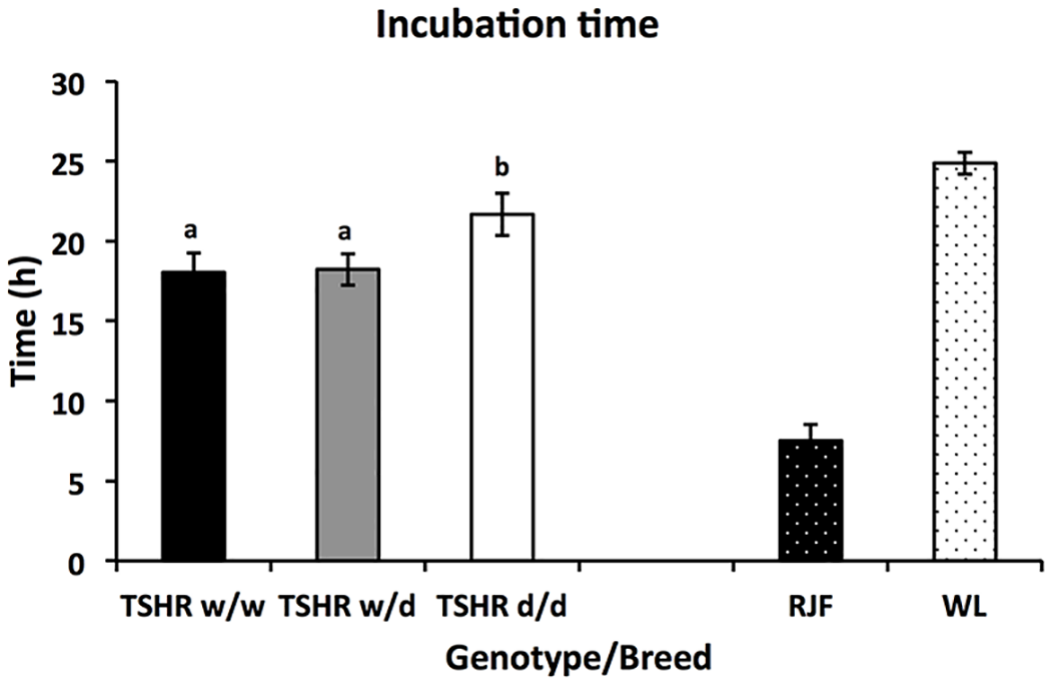
Estimated marginal means for incubation time (±SEM) from first hatch for each genotype/breed. TSHR genotypes: d/d: homozygous for the domestic allele, w/w: homozygous for the wild type allel, and w/d: heterozygous. RJF: Red Junglefowl, WL: White Leghorn. Post-hoc analyses *TSHR*: bars designated with different letters differ significantly at P<0.05.

Purebred WL chickens had a significantly longer incubation time than RJF (Fig 1) (Wald X^2^ (1) = 222.8, P<0.001). For *TSHR* chicken (which refers to the Locus Controlled Advanced Intercross Line birds, with a known genotype at the *TSHR* locus) there was a significant effect of genotype on incubation time (Wald X^2^ (2) = 3.8, P<0.001), with *d/d* genotypes hatching later.

The estimated marginal means, and SEM of the variables from the different behavioural tests are summarized in Table 2. For the Open Field test, there was a numeric difference between the purebred birds, in that RJF tended to spend more time in the start zone, but the effect did not reach significance (Wald X^2^ (1) = 0.119, P = 0.12). However, RJF moved significantly shorter distances than WL (Wald X^2^ (1) = 42.3, P<0.001). In *TSHR*-birds, there was likewise no significant effect of genotype on time spent in start zone (Wald X^2^ (18) = 22.4, P = 0.214), whilst there was a significant effect of sex, with males staying shorter (Wald X^2^ (1) = 4.85 P = 0.03), whilst *TSHR* genotype, as well as sex, had a significant effect on total distance moved, with no significant interactions between the two (Genotype: Wald X^2^ (18) = 33.5, P = 0–015; Sex: Wald X^2^ (1) = 5.8, P = 0.02; Genotype x Sex: Wald X^2^ (2) = 4.07, P = 0.13).

**Table 2:** Means (±SEM) of the variables measured in the open field (OF) and social reinstatement (SR) test.

	Open Field				Social reinstatement			
Genotype	Duration start zone (% time)		Total distance moved (cm)		Duration start zone (% time)		Total distance moved (cm)	
	Mean	SEM	Mean	SEM	Mean	SEM	Mean	SEM
***Wild type***								
RJF	19.2	5.9	1135.1	155.4	16.9	3.8	784.8	84.2
WL	9.7	1.5	3470.0	200.6	16.9	3.1	1125.4	81.0
***TSHR Females***								
w/w	16.1	8.1	3026.2	269.2	28.8	5.2	1605.2	154.3
w/d	19.1	4.1	2569.3	187.0	17.8	3.6	1692.3	107.4
d/d	12.7	5.9	2391.7	370.6	28.8	5.2	1427.5	201.9
***TSHR Males***								
w/w	48.9	6.9	1943.7	316.7	25.9	6.0	2049.1	178.6
w/d	20.4	5.0	1827.9	228.2	26.3	4.5	1847.2	132.7
d/d	12.1	5.8	2544.8	266.7	23.9	5.2	1779.9	152.9

P-values show the effect of genotype within each sex for TSHR chickens (Kruskal-Wallis) and effect of breed for both sexes for RJF/WL chickens (Mann-Whitney).

With respect to the Social Reinstatement test, there was no difference in time spent in start zone between purebred RJF and WL, whilst WL again moved a longer distance in total during the test (Wald X^2^ (1) = 6.0, P = 0.014). In the *TSHR*-birds, there was a significant effect of genotype on time spent in the start zone (Wald X^2^ (18) = 36.0, P = 0.007), but not on total distance moved (Wald X^2^ (18) = 22.4, P = 0.21). There was a significant effect of sex on total distance moved, but no interaction with genotype (Sex: Wald X^2^ (1) = 5.85, P = 0.016; (Genotype x Sex: Wald X^2^ (2) = 1.1, P = 0.57)

**Fig 2:**
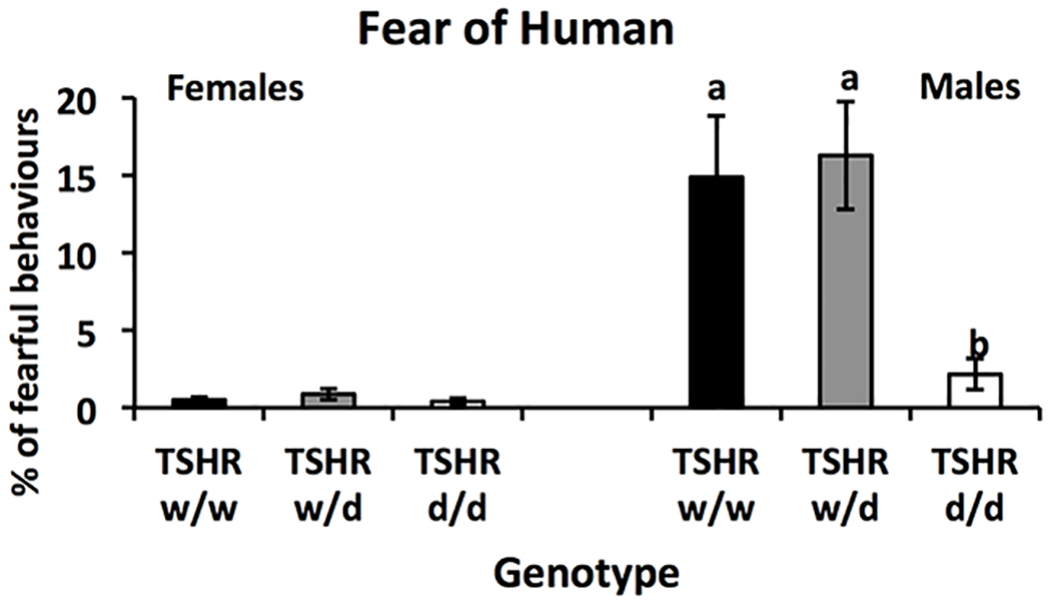
Average % of fearful behaviours in the fear of human-test (±SEM) for each genotype and sex of the *TSHR* chickens. Post-hoc analyses males: bars designated with different letters differ significantly at P<0.05.

In the Fear of Human-test, conducted on *TSHR*-birds, there was no overall effect of genotype (Wald X^2^ (18) = 15.3, P = 0.7), but a significant sex effect (Wald X^2^ (1) = 32.6, P<0.001) as well as a significant interaction between sex and genotype, where *d/d* males were less fearful (Wald X^2^ (2) = 10.8, P = 0.004) (Fig 2).

**Fig 3:**
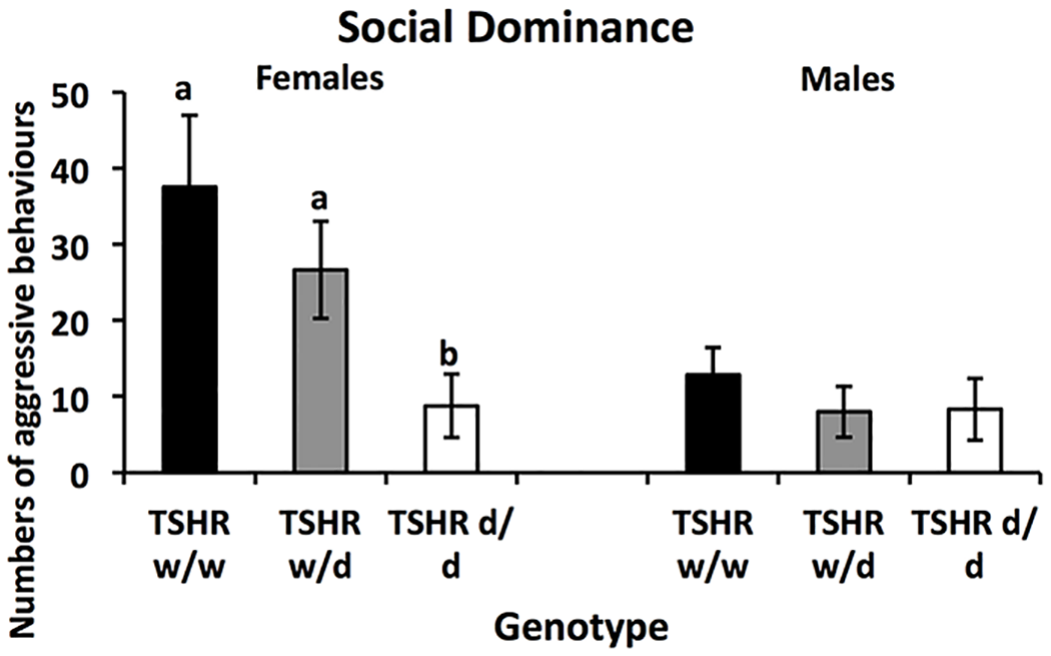
Average numbers of aggressive behaviours in the social dominance-test (±SEM) for each genotype and sex of the *TSHR* chickens. Post-hoc analyses females: bars designated with different letters differ significantly at P<0.05.

In the Social Dominance test, there was a significant effect of genotype (disregarding the nesting within family) (Wald X^2^ (2) = 71.5, P<0.001), of sex (Wald X^2^ (1) = 70.9, P<0.001), and their interaction (Wald X^2^ (2) = 26.5, P<0.001) (Fig 3). These effects were mainly caused by a lower frequency of aggression in *d/d* genotype females.

**Fig 4:**
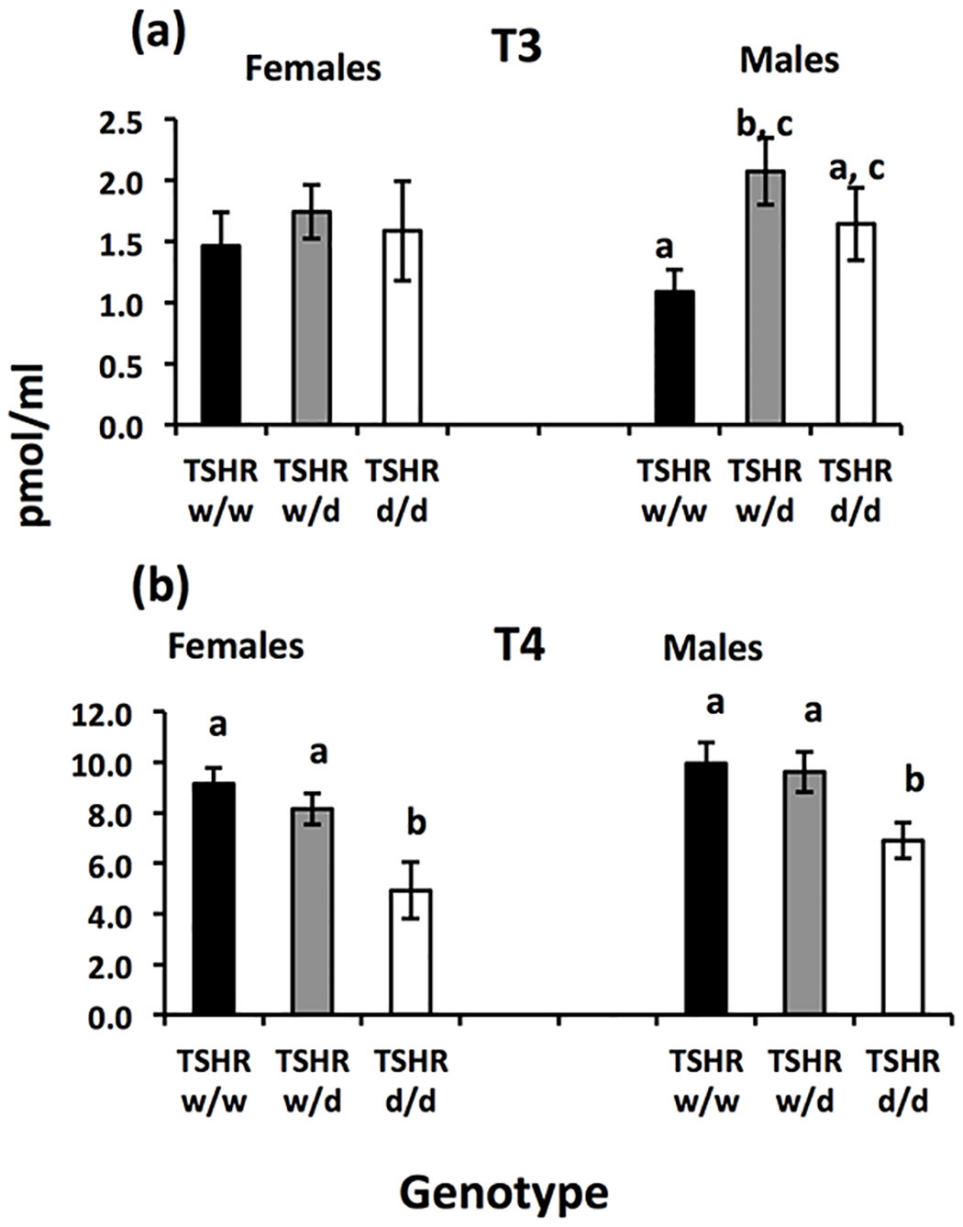
Average plasma concentration (±SEM) for each genotype and sex of the *TSHR* chickens. (a) Plasma T3 concentration (b) Plasma T4 concentration. Post-hoc analyses, T3 males and T4 females and males: bars designated with different letters differ significantly at P<0.05.

Plasma T3 and T4 levels are reported in Fig 4. There was a significant effect of genotype, but not sex or their interaction, on T3 (Genotype: Wald X^2^ (18) = 40.7, P = 0.002; Sex: Wald X^2^ (1) = 1.1, P = 0.28; Genotype x Sex: Wald X^2^ (2) = 1.9, P = 0.39). Post hoc analysis indicated that both heterozygous and *d/d* genotype males had higher levels than *w/w* genotype males. For T4, there was a significant effect on both genotype and sex, but no interaction, where d/d genotypes of both sexes had lower levels (Genotype: Wald X^2^ (18) = 33.5, P = 0.015; Sex: Wald X^2^ (1) = 5.8, P = 0.016; Sex x Genotype: Wald X^2^ (2) = 4.1, P = 0.13). Higher levels of both hormones were significantly associated with more aggression (T3: Wald X^2^ (6) = 38.0, P<0.001; T4: Wald X^2^ (6) = 222.8, P<0.001) but none of them with more fearful behaviour (T3: Wald X^2^ (7) = 10.8, P = 0.15; T4: Wald X^2^ (7) = 12.3, P = 0.09).

## Discussion

The results from this study showed that birds homozygous for the “domestic” allele (*d/d*) at the *TSHR* locus had a longer incubation time, *d/d* males showed less fearful behaviours in a fear of human-test, *d/d* females showed a lower number of aggressive behaviours in a social dominance-test, and both sexes showed a decreased levels of plasma T4. For incubation and the behavioural variables, the results of the TSHR-birds largely mirrored the differences between purebred RJF and WL. This indicates that the mutation in the *TSHR* gene [9] affects several traits associated with domestication, which supports the idea that this mutation has been positively selected. However, it is very likely that domestication in chicken has a polygenic basis as recently demonstrated for rabbit domestication [8] which suggests that the *TSHR* locus has significant but rather subtle phenotypic effect.

There was a difference between the *TSHR* genotypes in incubation time, where the *TSHR d/d* chickens had a significantly longer incubation time than *w/w* and *w/d* genotypes. This result followed the pattern from the parental RJF and WL chickens where the WL showed a significant longer incubation time than the RJF. TH levels increase during the second half of incubation with a pronounced peak in circulating T4 and T3 concentrations during the perihatch period [33]. The process of hatching is complex and THs are involved in many steps, like coordinating the growth and development of the “hatching muscle”, accelerating the onset of pulmonary respiration, retracting the yolk sac and initiate thermo regulation [18]. Hatching is delayed or even inhibited in embryos treated with different TH inhibitors. Furthermore, an administration of TH into the egg retracts the yolk sac and shortens the incubation time [18,33,34,35,36,37]. Based on findings from earlier studies it is therefore likely that the difference in incubation time seen in the *TSHR* chickens is caused by altered TH levels due to the mutation in the *TSHR d/d* genotype.

In the open field and social reinstatement tests, performed at a young age, the WL in general moved significantly longer than the RJF and spent less time in the start zone of the open field-test, which is in line with previous studies [5] and has been interpreted as a less fearful behaviour. The analysis of the effects of TSHR-genotype either mirrored the differences between the purebred birds, or showed no consistent differences. The reason for the relatively weak effect of genotype in the young birds may be that plasma TH concentrations in juvenile and adult birds are lower than during the perihatch peak [16]. However, in adults significant behavioural differences were seen in males in the fear of human-test, where the *d/d* genotype showed less fearful behaviours, and female *TSHR d/d* displayed a lower number of aggressive behaviours in the social dominance-test than the *w/d* and *w/w* genotypes. Overall, the pattern of *TSHR* genotype effects follows the observation that WL are less fearful [5] and less involved in social interactions [7]. This suggests that genotype on *TSHR* may play an important role in domestication effects on behaviour in chickens.

The results from this study show some clear sex differences on behaviours. The sex differences were most pronounced in the fear of human test, where males show higher levels of fearful behaviours than females, and in the social dominance test where females were more aggressive than males. Sex differences are common and one possible explanation could be epigenetic effects [38]. Another theory could be that selection pressure has been different for females and males during domestication. Females with low fear towards humans have possibly been favoured in the selection process for egg production traits, and males with low social dominance are likely better adapted to large groups with high density of animals.

Unfortunately, we were not able to measure TH levels in purebred WL and RJF chickens, and so far there are no data on TH levels in RJF available. Because of this, comparison of our TH-measurements with RJF/WL data is impossible. However, plasma T3 did not differ between *TSHR* genotypes in females. In the *TSHR* males the *w/w* genotype had a significantly lower level of T3 in comparison to the *w/d* but not the *d/d* genotype. However, a significant difference was seen for plasma T4 levels, where the *TSHR d/d* genotype showed significantly lower levels in comparison to the other two genotypes for both females and males, with the *w/d* genotype as intermediate.

There were strong effects of both T3 and T4 levels on aggressive behaviour, corroborating earlier findings in other species. T4 has been reported to social behaviour rats [21] and social stress can even induce hypothyroidism in intruders [39]. Moreover, hypothyroidism is strongly associated with emotional behaviour and depression [40] and with reduced exploratory behavior and hypoactivity [41–44], although the results are not always consistent [23,45–47]. In dogs, low TH levels have been shown to be associated with owner-directed aggression, where TH replacement is suggested as a possible therapy [20]. Although, we did not find any effects of hormone levels on fear of humans, it remains an exciting possibility that the *TSHR*-mutation studied here may exert an effect on domesticated chicken behavior by modifying T3 and T4 levels, since decreased social dominance and less fearful behaviour towards humans are characteristic in domesticated animals [1,2].

The domestication of the chicken started already 6000 B.C. [10] and the strong selective sweep with almost complete fixation of a variant allele at the *TSHR* locus indicates that the mutation is old, and may be a domestication locus in chicken [9]. However, a recent study by [11] suggested that the fixation of the variant allele at *TSHR* took place only in the past 500 years although the mutation was common already in Roman chickens 2,000 years ago. During modern time domestic chickens have mainly been bred for either meat or egg production. In the industry, breeders select for traits connected to reproduction, such as age of sexual maturity, rate of lay before and after moult and egg weight [48]. The thyroid system is known to be heavily involved in seasonal reproduction, regulating the release of follicle-stimulating hormone and luteinizing hormone from the pituitary gland, and it is activated during the breeding season resulting in a dramatic change in gonadal size [49]. It is possible that the *TSHR* mutation is related to the absence of seasonal reproduction, a classic feature of domestic animals, and has therefore been of selective advantage during the domestication of the chicken. The effect of the *TSHR* mutation [9] on seasonal reproduction remains to be investigated, but it seems likely that altered social and fearful behaviours could be a side-effect due to selection for a higher, non-season-dependent reproduction in chicken, possibly governed by a mutation in the *TSHR* gene. Another possibility could be that the gene is differentially expressed between genotypes in brain that further affects behaviour, or that it is not exclusively the TSHR gene that causes the phenotypic differences seen between genotypes. Genes within the same linkage disequilibrium (LD)-block as *TSHR* could differ between genotypes and hence explain the different phenotypic outcome. Within a 500 kb region around the gene (a putative LD-block), five genes in addition to *TSHR* are located. These five are: *DIO2* (type II iodothyronine deiodinase), *CEP128* (centrosomal protein), *GTF2A1* (general transcription factor IIA, 1), *STON2* (stonin 2: adapter protein involved in endocytic machinery), *SEL1L* (sel-1 suppressor of lin-12-like). From these *DIO2* is probably the most relevant gene because of its close relation to *TSHR*, by converting T4 to T3, and can hence affect phenotype. Differences in *DIO2* between *TSHR* genotypes are possible, and epistatic interaction between the two genes could contribute to the phenotypic differences seen in our study. However, this scenario rather supports the hypothesis that the HPT-axis has been targeted for selection during domestication and the strong selective sweep at the *TSHR* locus with an almost complete fixation over a 40 kb region strongly indicates the importance of this gene.

In conclusion, the present study shows that chickens homozygous for the mutant allele (*d/d*) at the *TSHR* locus show a longer incubation time, less fearful behaviour towards humans and less aggressive behaviour, compared to chickens homozygous for the wild type (*w/w*) allele or heterozygous birds (*w/d*). Furthermore, *TSHR d/d* chickens show a lower level of plasma T4 in comparison to the other *TSHR* genotypes, indicating that the mutation affects TH levels. Furthermore, the relationships between TH levels and aggression suggests that the mutation in *TSHR* may affect domesticated behavior through effects on plasma levels of T3 and T4. The prolonged incubation time and altered behavioural responses of the *TSHR d/d* in comparison to the *w/w* genotype mirrors the differences present in pure WL and RJF chickens. Hence, the mutation in *TSHR* affects typical domestication traits and may have contributed significantly to the evolution of the modern domestic chicken.

## Supporting Information

S1 Dataset (XLSX)
